# 

**Published:** 1897-10-23

**Authors:** 


### EARLY EDITION.

**Notices of Births, Deaths, and Marriages** are inserted in this column at a charge of 2s. 6d. for an announcement not exceeding four lines.

### NOTICE TO CORRESPONDENTS.

All MS., Letters, Books for Review, and other matters intended for the Editor should be addressed The Editor, "The Hospital," The Hospital Building, 28 & 29, Southampton Street, Strand, W.C.

The Editor cannot undertake to return rejected MS., even when accompanied by stamped directed envelope.

**Notice to Advertisers and Subscribers.**

All Advertisements, Orders for Copies of The Hospital, and Business Communications should be addressed to **THE MANAGER, (Not to The Editor),** The Hospital, The Hospital Building, 28 & 29, Southampton Street, Strand, London, W.C.

The Cost of Subscription, post free, is as follows:—Per week, 2½d.; per quarter, 2s. 8d.; per half-year, 5s. 3d.; per annum, 10s. 6d. Foreign:—Per annum, 15s. 2d.: payable, in all cases, in advance.

### SCRAPS AND GLEANINGS.

Nearly £200 has been raised by the Woking Hospital Saturday Fund.

The new wing of the Monkwearmouth and Southwick Hospital has been completed.

Up to the end of September only about £11,000 had been subscribed to the Cardiff Seamen's Hospital.

The Worshipful Company of Mercers have sent a donation of 50 guineas to the Royal Chest Hospital, City Road, E.C.

Bolton Hospital Saturday Fund collection for 1896-7, viz., £2,298, shows an increase of 12s. over that for 1895-6.

A portrait of the late Dr. W. J. Cleaver, one of the founders, has been presented to the Sheffield Children's Hospital.

The late Mr. Benjamin Piercy, of Marchwill Hall, Wrexham, has left a legacy of £200 to the Royal Alexandra Children's Hospital.

The annual Saturday Street Collection in aid of the Peterborough Infirmary brought in £32 this year, against £37 collected last year.

A total collection of £389 has been made by the Middlesbrough Hospital Saturday and Sunday Fund, compared with £391 collected in 1896.

After deducting expenses, £122 has been handed over to the Teignmouth Hospital as the result of the recent fête held on the grounds of the institution.

Mr. Rowland Maclaren, M.P., for many years the chairman of the Llanelly Hospital Committee, has been presented with an address on leaving the town.

Messrs. Reynolds and Branson have been awarded a bronze medal for their "Bandage Shoot," at the Earl's Court Exhibition, now being held in London.

Watford Hospital Saturday Committee have collected nearly £200 this year. Of this sum £100 has been allocated to the Watford District Cottage Hospital.

The ceremony of fixing the memorial-stone of the new children's wing of the West Norfolk and Lynn Hospital was performed by the Mayor of King's Lynn at the end of last month.

Dr. Hugh Ernest Fraser, of Inverness, has been appointed superintendent of the Dundee Royal Infirmary, in place of Dr. Nathan Raw, who has received an appointment at Liverpool.

A marble bust of the late Dr. Gilbert Ward was unveiled at the Thomas Knight Memorial Hospital, at Blyth recently. To Dr. Ward's energy the establishment of the hospital is in a great measure due.

The Hull Hospital Sunday Collection in 1896 shows a decrease of £19 when compared with 1895, although the number of collections made was the largest in the history of the movement. Altogether £765 was collected from 154 congregations.

The work in the in-patient department of the Ingham Infirmary, South Shields, showed an increase during the past year, 37 more in-patients being treated in 1896-7 than in 1895-6. Out-patients and casualties have decreased by 889.

At the Mirfield Memorial Cottage Hospital during the year ended June 30th last 105 patients were admitted. For the first time since its establishment the receipts equalled the expenditure, the figures being £577 and £551 respectively. The amount now owing to the bank is £301.

There have been 104 patients under treatment at the Devizes Cottage Hospital during the year just ended. The daily cost of patients and staff was 4s. 1d. per head, as compared with 3s. 10¾d. in the previous year. Each patient cost on an average £5 19s. 1d., against £5 11s. 6d. in 1895-6.

The work done at the Blackpool Hospital, which was opened in August, 1894, has been increasing in value year by year. In the first year 63 in and 27 out-patients were treated; in the second, 94 and 57 respectively; and in the third, viz., that up to August, 1897, the numbers were 151 and 139 respectively.

Out of the 764 cases which were treated in the three asylums in the Madras Presidency in 1896, 464 were Hindus, 154 Christians, 105 Mohammedans, and 41 belonged to other castes. The total cost per head at these asylums was Rs.162 11a. 8p. at the Madras Asylum, Rs.119 3a. 1p. at Calicut, and Rs.74 7a. 5p. at Vizagapatam.

Over 200 persons have subscribed to the endowment fund of the Teddington Cottage Hospital, the Diamond Jubilee celebration of that district, and £350 has been collected.

Since 1883 the number of in-patients treated at the Sheffield Royal Infirmary has increased by about 20 per cent., whilst the income has only increased by about 12½ per cent.

The total population during the year 1896 at the six asylums in the Bombay Presidency was 1,006, or 28 more than in the previous year. Of these 817 were males, and 189 females. The total cost per head ranged from Rs.262 1a. 5p. at Colaba (the European asylum), to Rs.98 14a. 11p. at Ahmedabad, and averaged Rs.168 1a. 4p. for the six asylums.

Although the governors of the Cork Street Fever Hospital, Dublin, had hoped that on the cessation of the small-pox epidemic of the previous years, that 1896-7 would have been a period of comparative rest, yet that year has witnessed a greater number of admissions than any previous year. Altogether 2,632 patients have been treated. This increase has been chiefly due to an epidemic of scarlet fever.

The total of the collections on Hospital Saturday at Wrexham is £118, a sum larger than that collected on any previous occasion.

In place of the street collection the Norwich Hospital Sunday and Saturday Fund organised this year a house to house collection, which has added to the receipts of the fund more than double the amount received last year from the street collection. The town was divided into 33 districts, based on the polling districts, each of which was placed under the supervision of a lady who was assisted by numerous helpers, no less than about 300 ladies being employed in the work.

The work of popularising the management of the Margate Cottage Hospital has at last been accomplished. The committee, no longer self-elected, has been strengthened by several new members, the medical staff has been increased from two to six, and the rules have been remodelled so that the rights of subscribers are recognised, and a date fixed for an annual general meeting to be regularly held. When we state that for 18 years no meeting of subscribers had been held, it will be seen that it was about time something should be done.

### The Student of Medicine.

### APPOINTMENTS.

A. G. R. Cameron, M.B., B.S.Durh., M.R.C.S.Eng., L.R.C.P.-Lond., D.P.H.Camb., Assistant Medical Officer to the Gore Farm Hospital, Metropolitan Asylums Board, appointed by the Government of India to act temporarily as Medical Officer in the Sanitary Department. J. E. H. Davies, M.R.C.S.Eng., L.R.C.P.Lond., appointed Medical Officer of Health to the Wrexham Rural District Council. S. B. De Butts, M.D.Brux., M.R.C.S., L.R.C.P., appointed Medical Officer to Messrs. J. Cook and Son's (Egypt) Nile Steamship Service. H. L. Ewens, M.D.Durh., M.R.C.S.Eng., appointed Medical Officer for the St. Pero District of the Maldon Union. Dr. W. F. McA. Hewling, appointed Medical Officer of the Third District of the Leicester Union. T. F. Long, M.R.C.S., L.R.C.P., appointed Medical Officer for the No. 8 District of the Aylesbury Union. S. McDonald, M.B., C.M.Edin., appointed House Surgeon to the Cumberland Infirmary, Carlisle. H. M. Morton, M.B., C.M.-Edin., appointed Assistant House Surgeon to the Cumberland Infirmary, Carlisle. J. H. Murray, M.B.Lond., M.R.C.S., L.R.C.P., appointed Assistant Medical Officer to the North-Eastern Fever Hospital, Metropolitan Asylums Board. W. S. Patch, M.B., appointed Junior House Surgeon to the Infirmary, Huddersfield. A. V. Peatling, L.S.A.Lond., appointed Clinical Assistant to Out patients at the Chelsea Hospital for Women, Fulham Road. S. Prior, M.B., appointed Non-resident House Surgeon to the Western Infirmary, Glasgow. J. G. C. Scott, M.B., appointed Medical Officer for the Brafield District of the Hardingstone Union. C. A. Seyler, B.Sc., F.I.C., F.C.S., appointed Public Analyst to the Glamorgan County Council. G. A. Shackel, M.R.C.S., L.R.C.P., appointed Medical Officer of Health to the Ludlow Rural District Union. W. Shirreffs, M.B., C.M.Aberd., appointed Medical Officer to the Employés of the Water Department of the Aberdeen Town Council. E. H. Snell, M.D.Lond., D.P.H.Camb., appointed Medical Officer of Health for the City of Coventry. F. Spicer, M.D.Durh., appointed Surgeon to the Metropolitan Ear, Nose, and Throat Hospital. Dr. F. H. A. Taylor, appointed Medical Officer for the Kidlington District of the Woodstock Union. P. Templeton, M.R.C.S., L.R.C.P., appointed Medical Officer for the Firbright District of the Guildford Union.

### ROYAL COLLEGE OF PHYSICIANS AND SURGEONS, EDINBURGH.

The following questions were set in the examination for the triple qualification :—

Materia Medica.—Five Years' Course.—(All the questions to be answered.)

1. Enumerate the "enemata" of the Pharmacopœia, and mention the proportion of the principal ingredient in each.
2. State the composition of pulv. scammon. co., mist. ferri co., pil. rhei. co., decoct. aloes co., liq. hydrarg. perchlor., and injec. morphinae hypodermica. (Omit proportion of ingredients in each.)
3. With regard to cinchona and galls, mention (a) their botanical origin, (b) their physical properties, (c) their active principles, and (d) their Pharmacopœial preparations and doses.
4. Write in unabbreviated Latin a prescription for a mixture containing potas. chlorat., tr. fer. perchlor, glycerini, spt. chlorof., aq.

